# Short Birth Intervals and Long-Term Stunting Risk in Indonesian Children: A Retrospective Cohort Study Using the Indonesian Family Life Survey

**DOI:** 10.3390/nu18142309

**Published:** 2026-07-14

**Authors:** Eflita Meiyetriani, Budi Utomo, Siti Dariyani

**Affiliations:** 1 Southeast Asian Ministers of Education Organization-Regional Centre for Food and Nutrition (SEAMEO RECFON), Pusat Kajian Gizi Regional (PKGR) Universitas Indonesia, Jakarta 13120, Indonesia; 2 Department of Population and Biostatistics, Center for Health Research, Faculty of Public Health, Universitas Indonesia, Depok 16424, Indonesia; budi.utomo.ui@gmail.com; 3 Doctoral Program in Public Health, Faculty of Public Health, Universitas Indonesia, Depok 16424, Indonesia; sitidariyani82@gmail.com

**Keywords:** birth spacing, child nutrition, high-risk fertility behaviors, stunting

## Abstract

**Background/Objectives**: Optimal birth spacing plays an important role in supporting children’s growth and nutritional outcomes. However, evidence on the long-term effects of short birth intervals on stunting from childhood to adolescence in Indonesia is limited. This retrospective cohort study used longitudinal data from waves 3, 4, and 5 of the Indonesian Family Life Survey (IFLS) (2000, 2007, 2014) to examine the association between short birth intervals and stunting trajectories among Indonesian children. **Methods**: We analyzed 1401 children aged 0–59 months with complete anthropometric data using longitudinal data from IFLS waves 3, 4, and 5 (2000, 2007, 2014). Stunting was defined as a height-for-age z-score (HAZ) below −2 standard deviations (SDs) according to the WHO Child Growth Standards (2006) for children under five years and the WHO Growth Reference (2007) for children and adolescents. The impact of short birth intervals on stunting status was evaluated using multilevel binary logistic regression. **Results**: Stunting distribution showed significant improvement between 2000 and 2014. In 2000, 37.7% of children were stunted, which decreased to 33.9% in 2007 and nearly 25% in 2014. The majority of mothers (77.7%) gave birth between 20 and 35 years of age, while 12.7% gave birth too early (<20 years) and 9.6% gave birth too late (>35 years). In addition, 16.2% of parents had too many children (parity ≥ 4), and 6.8% had too close birth intervals (<24 months). Multilevel logistic analysis revealed that short birth intervals (odds ratio (OR) = 1.60–1.63) were significantly associated with stunting after adjusting for child, household, socioeconomic, and environmental factors. However, maternal age and parity were not significantly associated with stunted growth. **Conclusions**: Birth spacing is a critical risk factor for stunting in early childhood and remains significant as children age. Longitudinal analyses highlight the importance of sustained interventions targeting maternal health, education, and child nutrition to address stunting in Indonesia.

## 1. Introduction

Stunting represents a critical global public health challenge, with particularly severe implications for low- and middle-income countries, including Indonesia. Impairment of linear growth due to prolonged nutritional deficiencies, recurrent infections, and inadequate psychosocial stimulation not only hinders individual physical and cognitive development but also significantly undermines national human capital and economic productivity [1,2,3,4]. Addressing stunting is pivotal for achieving sustainable development goals, particularly in fostering high-quality human resources, which are essential for global competitiveness.

Indonesia continues to grapple with high rates of stunting prevalence. While the 2022 National Nutrition Status Survey reported a decline to 21.6% from 24.4% the previous year, this figure remains above the WHO threshold of 20% and far from Indonesia’s national target of 14% by 2024 [5]. The persistent challenge of reducing stunting prevalence underscores systemic inadequacies in policy implementation and calls for multifaceted interventions addressing nutritional, socioeconomic, and maternal health determinants.

A study from the Demographic and Health Survey (DHS) in 45 countries found a significant positive relationship between mortality and malnutrition and specific fertility-related behavioral risk factors among children aged 5 years [6]. Maternal age at birth, short birth interval, high parity, unwanted pregnancies, and maternal morbidity (e.g., HIV/AIDS, pregnancy complications) exacerbate maternal and child health challenges and increase the risks for both the mother and child, leading to complications such as anemia, malnutrition, and stunting [7,8,9].

Among the maternal risk factors for stunting, pregnancies and childbirths classified under the “Four Too” framework—too young, too old, too close, and too many—warrant particular attention. The term “Four Too” refers to births in which the mother is too young or too old at the time of delivery (under 20 or over 34 years old), the birth interval is too short (less than 24 months), or the number of children is too many (more than three) [10,11,12]. Although these factors are recognized as important determinants of adverse birth and nutritional outcomes, evidence regarding their relative contribution to long-term linear growth of children remains limited, particularly in Indonesia. Therefore, this study examines the association between these high-risk fertility behaviors and stunting trajectories from early childhood to adolescence, with particular attention to identifying which factor exerts the most persistent influence over time.

Data from the Indonesia Demographic and Health Survey show that the percentage of high-risk births—including those falling under the “Four Too” category—declined from 42.8% in 1991 to 29.7% in 2017. However, a study in Indonesia revealed that approximately 40% of parents still experienced high-risk births [13]. These high-risk fertility behaviors are associated with adverse maternal and child health outcomes, including pregnancy complications, maternal anemia, and stunting [14,15].

Research has shown that children born after shorter intervals are more likely to experience poor nutritional outcomes [6,16]. Dewey and Cohen [16] analyzed 22 studies conducted since 1966 and found that longer intervals between births generally reduce the risk of malnutrition. Birth intervals of at least 36 months are associated with a 10–50% reduction in stunting in certain populations. This evidence underscores the importance of adequate birth spacing for improving the nutritional outcomes of children. A cross-sectional study from a city in Madhya Pradesh showed that the risk of stunting is approximately twice as high in children with a birth interval of <24 months compared to children aged 48+ months [17]. A study conducted in 34 Sub-Saharan African countries found that short birth intervals (<24 months) are associated with undernutrition and a 57% higher risk of infant mortality [18].

Maternal age also plays a crucial role in determining children’s health. Studies have found that younger maternal age, especially during early marriage and childbirth, increases the likelihood of child stunting [19]. In contrast, older parents (35–49 years) were more likely to have healthier children than younger parents (15–24 years), with significantly lower odds of stunting [20]. The challenges faced by young mothers, such as insufficient antenatal care, limited education, and psychological immaturity, can lead to low birth weight and hinder proper child-rearing practices [21,22]. In addition, issues such as insufficient breast milk supply may impede exclusive breastfeeding, contributing to stunting [23].

Higher parity significantly influences a child’s nutritional status. Children from larger families often face increased risks of stunting due to sibling competition, reduced maternal care, and the association between large family size and lower socioeconomic status [24,25]. For instance, a previous study found that children with more siblings in Sao Leopoldo, Brazil, had a higher likelihood of stunting, even after accounting for socio-demographic factors [25]. Similarly, having additional older or younger siblings negatively affected the arms of girls in Bolivia, highlighting the impact of family dynamics on nutrition [24].

As a growing country, Indonesia continues to face a substantial prevalence of unwanted and the “Four Too” births, underscoring the importance of family planning programs in addressing maternal health challenges. Reducing high-risk births through such programs can significantly decrease maternal mortality rates and improve child health outcomes. The high rate of high-risk births in low-income countries indicates an unmet need for family planning, the continued prevalence of child marriage, and a weak health care system [15].

The prevalence of unintended and the “Four Too” births in Indonesia from 2000 to 2017, as reported by the Indonesia Demographic and Health Surveys, showed a slight decline; however, this condition was still higher than the WHO target. The proportion of unintended births remained stable, ranging from 7.6% to 7.9% throughout the study period. In contrast, the prevalence of the “Four Too” births declined from 52.8% in 2002/3 to 43.5% in 2017. The prevalence of female child marriage in Indonesia was quite high, reaching approximately 12.53 [26]. This situation highlights the ongoing need for targeted interventions in Indonesia to address these risks and improve maternal and child health outcomes.

In Indonesia, the relationship between short birth intervals and stunting has been underexplored, particularly using robust longitudinal datasets. Previous studies primarily relied on cross-sectional data, such as the Demographic and Health Surveys (DHSs), which are limited in capturing long-term causal relationships. Longitudinal data from the IFLS, covering over 80% of the country’s population, provides a unique opportunity to assess the sustained impact of the “Four Too” births on stunting across childhood and into adolescence [27].

Based on the above background, this study will assess the impact of the “Four Too” births, especially short birth intervals, on stunting from three waves of the IFLS. This study will also examine the influence of other factors, such as socio-demographic, parental, and environmental factors, as potential confounders affecting stunting. This study provides evidence-based insights into the impact of high-risk births on stunting in Indonesia. By examining additional socio-demographic and environmental factors as potential confounders, the findings will support targeted interventions to reduce stunting prevalence and improve maternal and child health outcomes.

## 2. Materials and Methods

### 2.1. Study Design

This study employed a retrospective cohort design using data from the IFLS Survey (RAND Corporation, Santa Monica, CA, USA; SurveyMETER, Yogyakarta, Indonesia), a nationally representative longitudinal household survey. The IFLS is one of the country’s comprehensive population-based surveys that integrates information collected from households, individuals, and community facilities. Since its initiation in 1993, the survey has covered approximately 83% of the Indonesian population across 24 provinces, including Sumatra, Java, Bali, West Nusa Tenggara, Kalimantan, and Sulawesi. The survey’s fifth wave (IFLS-5), conducted in 2014, included approximately 15,900 households and more than 50,000 individuals. RAND Corporation and SurveyMETER jointly conducted the survey using standardized data collection procedures and extensive field supervision to ensure data quality [27].

This study focused on children aged 0–59 months identified in IFLS-3 (2000) and followed the same individuals longitudinally through IFLS-4 (2007) and IFLS-5 (2014), when they were approximately 7–12 and 14–19 years of age, respectively. The study employed a retrospective cohort approach by tracing eligible children across three surveys. Participant selection was based on predefined eligibility criteria rather than random resampling. Children who were biological children living with their parents, singleton births, alive throughout the observation period, and had complete anthropometric measurements available across the study waves. Children with biologically implausible anthropometric measurements, premature births, or those classified as SGA were excluded from the analysis.

Preterm and SGA infants were excluded because both conditions represent biologically distinct pathways to impaired growth that may differ from the postnatal nutritional and environmental mechanisms that were the primary focus of this study. Preterm infants often follow different growth trajectories compared with term infants, even in the absence of nutritional deprivation, while SGA may reflect intrauterine growth restriction or constitutional factors related to fetal development rather than postnatal exposures. In addition, the application of the WHO height-for-age standards may be less straightforward in these groups, particularly for preterm infants, who ideally require age correction during growth assessment.

**Figure 1:**
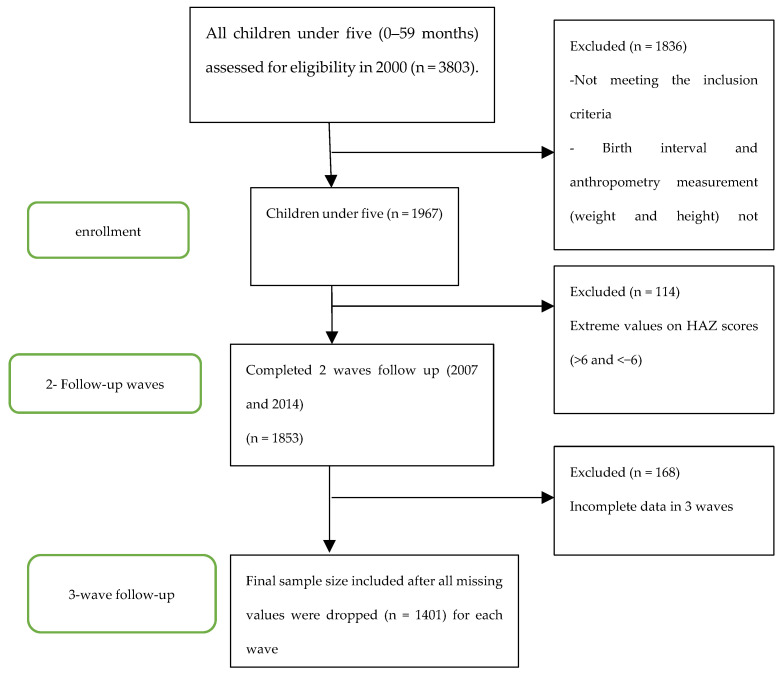
Flowchart of the respondents’ selection from IFLS waves 3, 4, and 5 (2000, 2007, 2014).

Based on a two-proportion formula, the minimum required sample size was estimated to be 1160 participants (see Appendix B for the sample size measurement), while the final analytical sample exceeded this requirement. Figure 1 presents the participant selection process.

Figure 1 shows the selection of respondents in this study.

Figure 1 presents the flow of the study participants. Of the 3803 children aged 0–59 months assessed for eligibility in 2000, 1836 (48.3%) were excluded because they did not meet the inclusion criteria or had incomplete data on birth interval or anthropometric measurements. Of the remaining 1967 children, 114 (5.8%) were lost to follow-up after the second wave (2007) due to attrition, and an additional 168 (8.5%) had incomplete data across the three waves, resulting in a final analytical sample of 1401 children (71.2% retention from the eligible cohort).

To assess the potential impact of attrition, the baseline characteristics of the children included in the final sample were compared with those of the children who were lost to follow-up. No significant differences were observed in sex (male: 52.9% vs. 51.8%, *p* = 0.62), maternal age (mean: 27.4 vs. 27.1 years, *p* = 0.45), or birth interval distribution (short interval: 6.8% vs. 7.2%, *p* = 0.58). However, children who were stunted at baseline were slightly more likely to be lost to follow-up (41.2% vs. 35.8%, *p* = 0.08), suggesting that our estimates of stunting prevalence in later waves may be modestly conservative. In addition, children from the lowest wealth quintile were somewhat more likely to be lost (24.5% vs. 22.1%, *p* = 0.09), which may have resulted in a slight underestimation of socioeconomic disparities in stunting.

### 2.2. Ethics Statement

This study is based on publicly available deidentified data. The use of the dataset for this study was approved by the Ethical Committee of the Faculty of Public Health, Universitas Indonesia (Number: Ket-572/UN2.F10.D11/PPM.00.02/2024, 12 September 2024). The Institutional Review Boards (IRBs) reviewed and approved the IFLSs and their procedures in the United States (at RAND) and in Indonesia at the UGM. Written informed consent was obtained from all respondents before data collection.

The IFLS collects extensive information on demographic characteristics, socioeconomic conditions, individual health, social capital, residential area infrastructure, and other variables. The IFLS dataset also includes comprehensive physical health assessments, which is a key strength of the survey. In the initial IFLS wave, anthropometric measurements (height and weight) were collected from all respondents. The range of health assessments was significantly expanded, starting with the IFLS2. Each household interview team included a trained nurse who measured the respondents’ height, weight, blood pressure, pulse, lung capacity, and hemoglobin levels. The respondents also performed a timed sit-to-stand test to evaluate physical functioning [27].

### 2.3. Variables and Operational Definitions

Table A1 (Appendix A) presents the variables and their operational definitions used in this study. The table categorizes the variables into dependent, main independent, and covariate variables, describing the definition, measurement approach, and data source of each variable. The outcome variable was stunting. Stunting was defined as a height-for-age z-score (HAZ) below −2 standard deviations (SDs) from the median of the WHO Child Growth Standards (2006) for children aged 0–59 months and the WHO Growth Reference (2007) for children and adolescents aged 5–19 years. Children with HAZ ≥ −2 SD were classified as non-stunted. All variables were derived from the IFLS datasets from 2000, 2007, and 2014, with the measurement and classification details provided in the table.

### 2.4. Data Quality and Bias Control

Several procedures were performed to ensure data quality and minimize potential bias sources. During data collection, the IFLS implemented rigorous interviewer training, standardized measurement protocols, and extensive field supervision. Anthropometric data were collected by trained nurses using calibrated instruments. The height-for-age z-scores (HAZ) were calculated using the WHO Anthro software (version 3.2.2; WHO, Geneva, Switzerland) for children aged 0–59 months and the WHO AnthroPlus software (version 1.0.4; WHO, Geneva, Switzerland) for participants aged 5–19 years. Observations with biologically implausible values (HAZ < −6 SD or >+6 SD) were excluded from the analysis according to the recommendations of the WHO.

Potential sources of bias were considered throughout the study. Selection bias was minimized using the nationally representative IFLS sample, which employed a multistage stratified sampling design covering diverse geographic and socioeconomic populations across Indonesia. Information bias was reduced through standardized questionnaires and direct anthropometric measurements performed by trained field personnel. Because the study used prospectively collected longitudinal data, recall bias was expected to be lower than that in conventional retrospective surveys. Confounding bias was addressed through multivariable modeling procedures that adjusted for child, parental, household socioeconomic, and environmental characteristics identified from previous literature and conceptual frameworks on stunting determinants.

Physical activity was assessed using a questionnaire adapted from the International Physical Activity Questionnaire (IPAQ), which measures the frequency and duration of moderate and vigorous physical activities during the previous 7 days. However, physical activity data were only available in the later survey waves (2007 and 2014), when participants were approximately 7–12 and 14–19 years old, respectively, and were not collected at baseline in 2000.

Physical activity was included as a time-varying behavioral covariate in the multilevel analysis to account for contemporaneous differences in activity levels across follow-up periods. Therefore, we did not treat physical activity as an early-life exposure or baseline determinant of stunting but rather as a concurrent behavioral factor that may be associated with growth status during later childhood and adolescence.

Missing data were evaluated before analysis. Of the 1853 children who completed follow-up across the three survey waves, 168 (9.1%) were excluded because of incomplete information on anthropometric measurements or key covariates. Because the proportion of missing data was 10%, complete-case analysis was performed for all subsequent analyses. The low proportion of missing observations was unlikely to materially affect the study findings’ validity. Therefore, the final analytical sample included 1401 children with complete longitudinal data from 2000, 2007, and 2014.

### 2.5. Statistical Analysis

Descriptive statistics were used to summarize the characteristics of the children, parents, households, and HRFs across the study period. Categorical variables were presented as frequencies and percentages, whereas continuous variables were presented as means and standard deviations or medians and interquartile ranges.

The primary outcome was stunting status, defined as a binary variable based on height-for-age z-score (HAZ) < −2 standard deviations according to the WHO Child Growth Standards and WHO Growth Reference. The data had a hierarchical structure in which measurement occasions were nested within children and children were nested within households because repeated observations were available for the same child across three survey waves (2000, 2007, and 2014).

Multilevel mixed-effects logistic regression models were fitted to account for clustering and within-subject correlation. Random intercepts were specified at the household and individual child levels to accommodate unobserved heterogeneity and repeated measurements over time. Model building was conducted sequentially. The null model only included random effects to assess the extent of clustering. Subsequently, the following variables were entered in blocks according to a conceptual framework: (1) high-risk fertility behavior variables (maternal age at childbirth, parity, and birth interval), (2) child characteristics, (3) parental characteristics, (4) household socioeconomic and environmental factors, and (5) behavioral factors. Adjusted odds ratios (AORs) and 95% confidence intervals (CIs) were calculated.

The model fit was assessed using the LR test and log likelihood statistic. The contribution of clustering was evaluated using the intraclass correlation coefficient (ICC) and the MOR. The ICC quantified the proportion of total variance attributable to household and individual levels, whereas the MOR translated between-cluster variation into an OR scale to facilitate interpretation.

Before multivariable analysis, collinearity among explanatory variables was examined using variance inflation factors (VIFs). Variables identified from previous literature and theoretical considerations as potential confounders were retained in the adjusted models regardless of statistical significance. Missing data were assessed before analysis. Because the proportion of incomplete observations was below 10% (9.1%), complete-case analysis was considered appropriate and applied in all regression models. Statistical significance was determined at a two-sided *p*-value < 0.05. Stata version 16 (StataCorp LLC, College Station, TX, USA) was used for all statistical analyses.

## 3. Results

### 3.1. Child Characteristics

The study population comprised children aged 0–59 months with complete anthropometric data across three IFLS waves. A descriptive analysis of the characteristics of the children was conducted before analyzing the role of the “Four Too” in linear growth (Table 1).

Table 1 presents the characteristics of the children in this study based on the baseline data (wave 3 in 2000). The majority of children (33%) were aged 12 to 24 months. Males slightly outnumbered females (52.9% vs. 47.1%). Over 94% had normal birth weight, whereas 5.5% had low birth weight. Preterm birth prevalence was 9.0%. Early breastfeeding initiation was universal (98.1%), but exclusive breastfeeding rates were low (9.1%). ARI (69.7%) and infectious diseases (77.0%) were common, whereas 17.8% reported diarrhea. Most births (51.1%) occurred at home or with traditional attendants, and 51.7% of deliveries were assisted by midwives.

Table 2 shows that between 2007 and 2014, the proportion of children with poor dietary diversity (FCS < 35) nearly doubled (18.6% to 36.3%) between 2007 and 2014, while physical activity improved (inadequate levels decreased from 77.6% to 56.7%). Smoking data (2014 only) showed that 9.9% of adolescents smoked.

### 3.2. Parental and Household Socioeconomic Characteristics

Table 3 shows that the levels of parental education varied, with most parents having low education (≤junior high school). The socioeconomic status of households was assessed using food expenditure ratios and asset ownership. Urban residence increased from 45.5% in 2000 to 62.0% in 2014. Households with poor food expenditure ratios (≥60%) decreased from 63.2% to 22.6%, reflecting improved economic conditions. Improved sanitation and drinking water access also increased significantly over time.

### 3.3. High-Risk Births (The Four Toos)

Table 4 shows that the majority of mothers (77.7%) gave birth between 20 and 35 years of age, while 12.7% were too young (<20 years) and 9.6% were too old (>35 years). In addition, 16.2% of parents had too many children (parity ≥ 4), and 6.8% had too close birth intervals (<24 months). Further analysis revealed that 36.1% of parents had one high-risk factor and 8.7% had two or more, increasing the risks of pregnancy complications and adverse child health outcomes.

### 3.4. Prevalence of Stunting in 2000, 2007, and 2014

**Figure 2:**
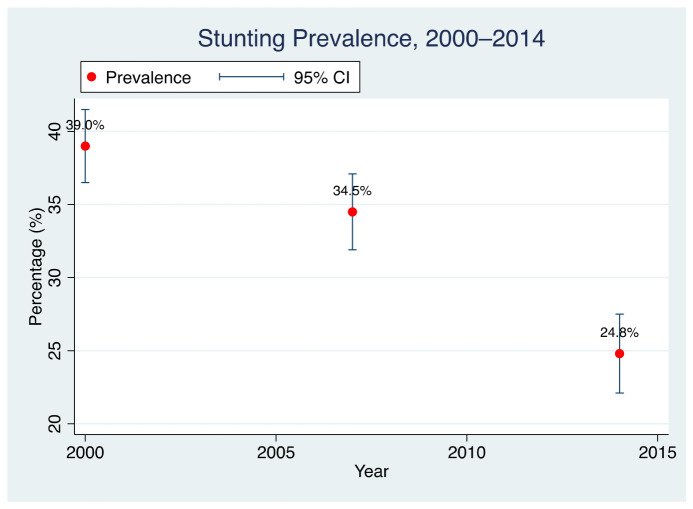
Stunting prevalence in 2000, 2007, and 2014.

The analysis presented in Figure 2 reveals a declining trend in the prevalence of stunting as the respondents grew older. In 2000, the prevalence of stunting reached 39%, which decreased to 35% in 2007 and further dropped by 10–25% in 2014. These findings indicate a persistently high prevalence of stunting among respondents aged 5 years in 2000. The high rate of stunting at the beginning of the observation period reflects suboptimal nutritional interventions during early childhood, particularly within the first 1000 days of life. Although a downward trend is evident, the prevalence of stunting in 2014, when respondents reached adolescence (aged 10–19 years), remained alarming.

To further clarify the observed decline in stunting prevalence across follow-up waves, we conducted an additional transition analysis examining individual-level changes in stunting status between 2000 and 2007, 2007 and 2014, and across the full follow-up period (2000–2014) (Appendix C, Table A2). The results showed that the stunting status was dynamic over time. Between 2000 and 2007, 21.9% of children remained stunted, while 17.0% experienced catch-up growth. Between 2007 and 2014, persistent stunting decreased to 13.1%, although 9.4% of children experienced height faltering. Across the entire follow-up period, 21.3% of patients remained stunted, whereas 25.4% achieved catch-up growth. Although the prevalence of stunting declined over time, a substantial proportion of children continued to experience persistent or recurrent growth faltering.

### 3.5. The Impact of the Four Toos Births on Stunting

Covariates were selected in this multivariate analysis based on the conceptual frameworks of stunting determinants and prior empirical evidence. Child factors (age, sex, and low birth weight) were included because they represent the biological determinants of growth. Parental factors (maternal height, parental education, and employment) were included to capture the intergenerational transmission of nutritional status and socioeconomic influences. Household factors (residence, sanitation, water source, asset quintile, and food expenditure ratio) were included to reflect environmental and socioeconomic conditions. Behavioral factors (physical activity and smoking) were included to assess lifestyle influences on growth outcomes.

The fixed-effects analysis (Table 5) revealed that short birth intervals (<24 months), one of the “Four Too” high-risk birth factors, consistently increased the likelihood of child stunting across the models (OR = 1.59–1.63; *p* < 0.01). Among child-related variables, older age groups (12–59 months) exhibited a significantly higher risk of stunting than younger children (odds ratio > 1; *p* < 0.01). Low birth weight was also strongly associated with increased odds of stunting (odds ratio (OR) = 1.55–1.69), emphasizing the importance of early-life nutritional and health conditions.

In terms of parental characteristics, a maternal height of 150 cm was a significant risk factor for stunting (OR ≈ 1.50), indicating the intergenerational transmission of nutritional disadvantage. Socioeconomic conditions further contributed to the risk: children living in households with poor sanitation (odds ratio [OR] = 1.33–1.40) and high food expenditure (i.e., >60% of total household spending allocated to food) (OR = 1.37–1.40) were more likely to be stunted. Conversely, residing in urban areas appeared to be protective (OR = 0.77), likely due to better access to services and infrastructure. Regarding behavioral factors, children in households with low physical activity levels showed a moderately increased risk of stunting (odds ratio [OR] = 1.21–1.23), while parental smoking did not have a statistically significant effect.

The random effects analysis and intraclass correlation coefficients (ICCs) were used to highlight the hierarchical data structure. Approximately 8–12% of the variance in stunting was attributable to household-level differences and 13–19% to individual-level differences within households. The MOR declined from 2.22 in the null model to 1.70 in the fully adjusted model, indicating that the inclusion of covariates accounted for part of the between-household heterogeneity. Nevertheless, the remaining unexplained variance demonstrates the influence of additional unmeasured contextual factors. Model diagnostics showed a significant improvement in log likelihood values (from −2630.38 to −2501.67), confirming a better model fit as more covariates were included. The LR tests yielded statistically significant results (*p* < 0.001), indicating that the added variables enhanced the model’s explanatory power.

## 4. Discussion

This study provides critical insights into the determinants of stunting among children in Indonesia, with an emphasis on how factors evolve as children age. Although the prevalence of stunting significantly decreased from 37.7% in 2000 to approximately 25% in 2014, the analysis revealed varying predictors of stunting across different age groups and datasets. During early childhood, rapid physical growth demands high nutritional intake, and stunting often results from inadequate nutrition during this critical period [28]. However, as children enter adolescence, growth rates stabilize, and there is a greater opportunity for catch-up growth, particularly when improved nutrition and health interventions are introduced [28].

Adolescence represents the second critical window in a child’s life course for growth and development. The adolescent growth spurt offers an opportunity for recovery during this phase, especially for children previously affected by stunting. Nonetheless, the extent to which this window is used depends heavily on the availability of adequate nutrition, GHs, and a supportive environment. Unfortunately, many adolescents in low- and middle-income countries still face health disparities and often do not receive adequate nutritional interventions during adolescence [29].

A longitudinal study involving 7128 children aged 1–15 years in four countries (Ethiopia, India, Peru, and Vietnam) demonstrated that stunting is not confined to the first 1000 days of life. Instead, it may emerge, improve, or recur throughout adolescence. Approximately 30% of children experienced stunting for the first time at age one, and most remained stunted until age 15. However, some children recovered and later became stunted again, whereas others experienced stunting for the first time after the age of eight [30].

Moreover, the age at which stunting first occurs significantly influences subsequent growth patterns. The younger the age at exposure and the sooner it is addressed, the greater the likelihood of achieving normal growth or catch-up. However, stunting may persist into adolescence and even adulthood if left unaddressed. Therefore, a life-course approach is essential—starting from maternal nutrition during pregnancy, early childhood, school age, and continuing through adolescence—to prevent and address stunting.

A study of pregnant women and their children in rural Bangladesh yielded noteworthy findings. This study analyzed linear growth and stunting from birth to age 10 and investigated whether maternal and environmental conditions at conception were associated with linear growth during childhood and stunting at age 10. Results showed that the height-for-age z-scores (HAZ) declined until age 2 and then increased up to age 10, whereas the height-for-age difference (HAD) from the World Health Organization reference standards continued to widen up to age 10. The highest stunting prevalence was observed at age 2 (50%) and declined to 29% by age 10 [31].

The additional transition analysis (Appendix C, Table A2) provides important insights into the mechanisms underlying the observed decline in the prevalence of stunting from 39% in 2000 to 25% in 2014. The results reveal that stunting status was highly dynamic over the follow-up period, with substantial individual-level transitions occurring in both directions.

Between 2000 and 2007, 17.0% of children experienced catch-up growth (transitioning from stunted to normal), whereas 21.9% remained persistently stunted. Between 2007 and 2014, the proportion of children with persistent stunting declined to 13.1% between 2007 and 2014, although 9.4% of children who were previously normal experienced height faltering. Across the entire follow-up period (2000–2014), 25.4% of the children achieved catch-up growth, while 21.3% remained stunted. These findings indicate that the observed reduction in prevalence reflects individual recovery through catch-up growth, particularly during the transition from childhood to adolescence.

Several mechanisms may explain these longitudinal patterns. First, the decline in stunting prevalence likely reflects broader improvements in living conditions and socioeconomic circumstances that occurred in Indonesia during the study period. As shown in Table 4, urban residence increased from 45.5% to 62.0%, household food expenditure improved markedly (the poor food expenditure ratio decreased from 63.2% to 22.6%), and access to improved sanitation and drinking water increased substantially. These improvements reflect Indonesia’s wider economic development, poverty reduction, and investments in public health infrastructure during the 2000–2014 period.

Second, improvements in health care access and nutrition programs may have supported better child growth outcomes over time. The expansion of maternal and child health services, including antenatal care, skilled birth attendance, immunization programs, and growth monitoring, likely contributed to improved nutritional status. Additionally, national nutrition-specific interventions, such as the provision of micronutrient supplements and promotion of optimal infant and young child feeding practices, may have played a role in reducing the prevalence of stunting [6].

Third, the occurrence of height faltering among some previously normal children (9.4% between 2007 and 2014) highlights the vulnerability of children to recurrent growth setbacks during adolescence. This may reflect nutritional challenges during periods of rapid growth, increased nutrient demands, and the influence of environmental and behavioral factors during adolescence.

Fourth, selective loss to follow-up may have contributed modestly to the observed decline, as children with poorer baseline nutritional status were slightly more likely to be lost from the cohort (41.2% vs. 35.8%, *p* = 0.08), potentially leading to a modest underestimation of stunting in later waves.

Finally, the possibility of adolescent catch-up growth should be considered. Although the growth potential during adolescence is more limited than that during early childhood, improved nutrition and health conditions during this period may enable some individuals to recover from earlier growth deficits. However, the persistence of stunting in 21.3% of children across the full follow-up period indicates that early-life growth impairment has lasting consequences that extend into adolescence for many children.

In this study, female children exhibited significantly lower odds of stunting than male children (odds ratio = 0.82, 95% CI, 0.69–0.96). This finding is consistent with those of previous studies demonstrating sex-based disparities in early childhood child growth and nutritional outcomes [32,33]. Several biological mechanisms may account for this association. Male children generally experience more rapid growth and consequently have greater energy and nutrient requirements, rendering them more susceptible to nutritional deficits and adverse environmental exposures. Furthermore, evidence highlights that boys are biologically more vulnerable to infections and other health-related stressors during the critical growth and development periods, which may increase the risk of growth faltering [34]. In addition to biological factors, contextual influences, such as differences in caregiving practices, feeding behaviors, and intrahousehold resource allocation, may further contribute to the observed sex differences in stunting risk. Collectively, these findings support the notion that both biological susceptibility and social determinants affect shaping children’s growth trajectory.

The findings of this study challenge the prevailing literature regarding the association between births of the “Four Too” and stunting in children and adolescents. Unlike several previous studies, our analysis found no significant associations between maternal age, parity, and stunting risk. These results contradict numerous studies that have consistently highlighted the detrimental effects of these factors on child growth and nutrition. For instance, research in Sub-Saharan Africa has demonstrated that older maternal age (≥34 years) is a protective factor against child undernutrition, whereas younger maternal age increases the risk of adverse nutritional outcomes [17]. Studies in Ethiopia and Nigeria have emphasized the compounded risk of stunting in high-risk births, particularly in cases of short birth intervals and young maternal age [35,36].

The associations among maternal age, parity, and stunting in our fully adjusted models warrant careful interpretation. Several factors may explain why these variables lost significance after adjusting for birth spacing and other covariates. First, the inclusion of birth interval as a covariate may have captured much of the effect of parity, as women with higher parity are more likely to have shorter birth intervals due to the accumulated effect of multiple pregnancies. This collinearity between parity and birth interval may reduce the model’s independent contribution of parity. Second, the long follow-up period (14 years) and improvements in maternal and child health services may have attenuated the effects of maternal age and parity on child growth outcomes. Increased access to antenatal care, nutritional supplementation programs, and health education may have mitigated the risks associated with young or advanced maternal age. Third, unmeasured confounding factors, such as maternal nutritional status during pregnancy, may mediate the association between maternal age and stunting. Fourth, the relatively small number of children born to mothers in the “too young” (12.7%) and “too old” (9.6%) categories may have limited statistical power to detect significant effects. Finally, the Indonesian context may differ from other settings where maternal age and parity were significant predictors. Extended family support systems and community-based health programs in Indonesia may buffer some risks associated with young maternal age or high parity. However, the consistent association between short birth intervals and stunting across all models indicates that birth spacing is the most robust high-risk fertility behavior predictor in this population. These findings highlight the need for targeted interventions focused on promoting optimal birth spacing, while also recognizing that maternal age and parity remain important considerations for comprehensive maternal and child health programs.

The results of the analysis indicate that births occurring at very short intervals (<twenty-four months) are consistently associated with a significantly increased risk of stunting. In the multilevel model, children born less than 24 months after a previous sibling were 1.59–1.63 times more likely to be stunted than those with longer birth intervals, even after adjusting for child, parental, socioeconomic, and environmental factors. In contrast, maternal age at childbirth (either very young or older) and high parity (≥4 births) were not significantly associated with STD. These findings are consistent with those of previous studies, which have shown that short birth intervals may lead to maternal nutrient depletion, insufficient postpartum recovery, and increased caregiving burden [16,37]. Limited maternal attention and resources available for younger children can directly affect their dietary intake and health care. Thus, effective family planning and adequate spacing between pregnancies are essential strategies for preventing long-term stunting.

In East Africa, short birth intervals significantly increased the risk of both stunting and anemia [15]. Similar results were reported in a multilevel analysis that examined data from 32 sub-Saharan African countries and found that childbirth before age 18, high parity, and short birth intervals were significantly associated with stunting, wasting, and underweight. Interestingly, maternal age over 34 years at childbirth was negatively associated with underweight, demonstrating that older maternal age may be protective in certain contexts [18].

Longitudinal data from the YLS across four low- and middle-income countries demonstrated a consistent positive association between longer birth intervals and children’s improved height growth. Children born after short intervals tended to experience early growth faltering but showed potential for compensatory growth, particularly among boys [38]. These findings emphasize that while early health and nutrition interventions are crucial, they may not fully offset the negative impacts of the “Four Too” birth conditions.

Moreover, the discrepancy may be attributable to population heterogeneity and temporal differences in data collection. As our study spans 14 years, socioeconomic improvements and policy changes during this period could have reduced disparities and obscured the direct effects of maternal factors. Interventions promoting maternal education, antenatal care, and nutritional supplementation may have lessened the impact of traditional risk factors, such as high parity or short birth intervals.

Although previous cross-sectional studies have documented the association between short birth intervals and stunting, our longitudinal analysis provides unique insights into the persistence of this effect over time. By tracking the same children from infancy through adolescence, we demonstrate that the harmful impact of short birth intervals on linear growth extends into adolescence and is not limited to early childhood. This finding underscores the importance of addressing birth spacing as a long-term nutritional determinant rather than a short-term risk factor. Furthermore, our comprehensive adjustment for multiple confounding factors allows for a more precise estimation of the independent effect of BS, disentangling it from other correlated risk factors such as socioeconomic status and maternal characteristics.

Other maternal factors, such as maternal height, were identified as significant predictors of stunting. Short maternal stature is a known risk factor for stunting, which may reflect poor childhood nutrition or chronic infections [39]. The association between maternal height and stunting in children has both biomechanical and biological plausibility. Shorter women are more likely to have a narrower pelvis, which increases the risk of cephalopelvic disproportion and obstructed labor, which can result in adverse birth outcomes. In addition, shorter stature may reflect inadequate nutrient supply during maternal development, leading to limited nutritional reserves that may compromise fetal growth [40].

The intergenerational transfer of socioeconomic adversity further supports the association between maternal height and stunting. Maternal stature can serve as a marker of early-life nutritional and environmental stressors, reflecting prolonged exposure to poor nutrition and infections. These adversities not only impact maternal growth but may also influence offspring health through a cascade of biological and socio-environmental mechanisms. Chronic inflammation from infections and limited nutrient supply may impair metabolic processes during maternal development, diverting resources away from growth and contributing to long-term growth retardation [39,41].

The findings of this study indicate that the age group of children (12–24 months and 25–36 months) is significantly associated with the risk of stunting. These findings align with existing evidence showing that the risk of stunting increases as children age, particularly during the transition from EB to CF. The first 1000 days of life, spanning from conception to a child’s second birthday, are widely regarded as a critical window for laying the foundation for a child’s health and development. Nutritional intake, caregiving practices, and environmental factors play an essential role in determining growth and development outcomes [42]. Key practices, such as early breastfeeding initiation and exclusive breastfeeding for the first 6 months, significantly contribute to a child’s nutritional status. Moreover, other health behaviors, including healthy dietary practices and proper hygiene, are crucial in ensuring optimal child health and nutrition.

Optimal nutrition during the first 1000 days, often referred to as the “golden period,” is particularly important for brain growth and overall development. This phase includes critical periods such as pregnancy (280 days), 0–6 months of exclusive breastfeeding (180 days), and 6–24 months of complementary feeding (540 days). During this critical period, malnutrition can have irreversible consequences on a child’s physical and cognitive development, underscoring the importance of adequate nutrition and care during this time [43].

Several studies support the critical importance of this period. A study highlighted that inadequate complementary feeding practices starting at six months contribute to increased stunting risk [14]. Similarly, another study emphasized the heightened nutritional needs and vulnerability to infections during the transition from EB to CF, particularly in low-income settings.

A study in Sub-Saharan Africa found that children aged 12–23 months faced a significantly higher risk of stunting compared to younger age groups, primarily due to unmet nutritional demands during this rapid growth phase [28]. Targeted interventions, such as improving access to nutrient-rich complementary foods and educating caregivers about feeding practices, can reduce the prevalence of stunting [38]. In conclusion, the age group variable highlights the need to focus on the 1000-day period to prevent stunting and ensure optimal growth and development. Addressing nutritional and health needs during this window can have a profound impact on reducing stunting rates and improving the long-term health outcomes of children.

The consistent association between low birth weight and stunting across all multivariable models (OR, 1.55–1.69) underscores the critical importance of fetal growth for subsequent linear growth of the child. Notably, this association remained robust even after adjusting for birth spacing, socioeconomic factors, and other confounders, suggesting that LBW exerts an independent and persistent effect on growth outcomes that extends well beyond the early-life period.

Several biological mechanisms may explain this association. Infants born with low birth weight typically begin life with reduced nutritional reserves, including lower body fat and glycogen stores, which increase their vulnerability to growth faltering during periods of nutritional stress [44]. In addition, impaired organ development, particularly of the gastrointestinal, immune, and endocrine systems, may compromise nutrient absorption, increase susceptibility to infections, and disrupt growth hormone-insulin-like growth factor-1 (IGF-1) axis function, all of which can impair linear growth. Altered metabolic programming resulting from IUG may also predispose these children to metabolic inefficiencies that limit their capacity for catch-up growth, even when nutritional conditions improve [29].

The persistence of this association into adolescence suggests that the effects of suboptimal fetal growth may have long-lasting consequences that shape growth trajectories well beyond infancy and early childhood. This finding supports the life-course perspective of undernutrition, in which adverse conditions during fetal development can have lasting consequences for growth and health. Low birth weight may serve as an important biological link between maternal health and child stunting, reflecting maternal undernutrition, poor pregnancy health, and inadequate intrauterine growth. Moreover, LBW may conceptually interact with other early-life risk factors, including short birth intervals, inadequate infant feeding practices, and poor environmental conditions, potentially compounding the risk of persistent growth faltering.

The strong and consistent role of low birth weight in our study highlights the intergenerational cycle of undernutrition. Children born with low birth weight are more likely to become stunted in childhood, remain shorter in adolescence, and eventually enter adulthood with compromised nutritional status, increasing the risk of adverse birth outcomes in the next generation [45]. Breaking this cycle requires integrated interventions that strengthen maternal nutrition before and during pregnancy, promote adequate birth spacing, and improve child nutrition and health services during the critical early years of life. Our findings suggest that interventions targeting low birth weight prevention, including maternal nutritional supplementation, improved antenatal care, and management of maternal infections, could have substantial and sustained benefits for reducing stunting prevalence across the life course.

In addition to other factors, the analysis revealed that certain household factors also had a significant impact. The analysis demonstrates a significant association between unimproved sanitation and stunting in children. This indicates that children living in households with unimproved sanitation facilities are 35% more likely to experience stunting than those with improved sanitation facilities. These findings are consistent with those of previous studies that highlighted the critical role of adequate sanitation in child health and nutritional outcomes [46,47].

Unimproved sanitation increases fecal contamination exposure, leading to higher rates of diarrheal diseases and environmental enteropathy, both of which impair nutrient absorption and contribute to growth faltering [48]. Research supports this relationship, reporting a significant reduction in stunting prevalence when improved water and sanitation are ensured [46]. The results emphasize the need for integrated interventions addressing water, sanitation, and hygiene (WASH) to mitigate stunting. Policies promoting access to improved sanitation, alongside health education about hygiene practices, can play a vital role in breaking the cycle of poor sanitation and undernutrition, particularly in low-resource settings.

The association between low physical activity and increased odds of stunting (OR ≈ 1.21) should be interpreted with considerable caution due to the data’s temporal limitations. Because physical activity was measured only during later childhood and adolescence (2007 and 2014), it cannot be considered an antecedent determinant of early-life stunting. Instead, it may reflect a concurrent correlation of nutritional status or broader socioeconomic and environmental conditions that influence both physical activity levels and growth outcomes.

It is also plausible that the relationship is bidirectional. Children with stunting may have lower physical capacity, reduced muscle strength, lower energy reserves, and reduced exercise tolerance, which could limit their participation in physical activity and contribute to a sedentary lifestyle [29]. Conversely, physical activity during adolescence may influence body composition, bone density, and metabolic health, although its direct effect on linear growth (height) is less well-established and likely minimal compared with the effects of nutrition and genetics during the critical early-life growth period.

Therefore, the observed association likely reflects a complex interplay between growth status and behavioral adaptation rather than a direct causal pathway from physical activity to linear growth impairment. Future studies with repeated measures of physical activity beginning in early childhood are needed to clarify the temporal direction and potential causal mechanisms underlying this association.

Our findings indicate that among the “Four Too” fertility risk factors, only short birth interval remained significantly associated with stunting after adjustment for child, parental, household socioeconomic, and environmental characteristics. The stronger and more persistent effect of short birth spacing may reflect nutritional depletion, inadequate recovery between pregnancies, and competition for household caregiving and nutritional resources during the critical early-life period. In contrast, the effects of maternal age and high parity may be more indirect and may be mediated or attenuated by socioeconomic improvements and health care access. These findings confirmed that birth spacing should be prioritized within maternal and child nutrition strategies, while broader reproductive health interventions remain relevant for improving overall maternal and child well-being.

The absence of significant associations between maternal age at childbirth and parity with stunting after adjusting for birth spacing and other covariates is an important finding that warrants careful consideration and contextual interpretation. Although these variables are central components of the Four Too framework and have been consistently associated with child undernutrition in many settings [7,44], our results suggest that their influence on child linear growth may be less direct or may operate through other intermediary pathways in the Indonesian context.

Several contextual factors may explain why the findings from Indonesia differ from those reported in other settings, particularly in Sub-Saharan Africa and South Asia. First, over the past two decades, Indonesia has made substantial progress in expanding maternal and child health services. The proportion of births attended by skilled health personnel increased from approximately 60% in 2000 to over 90% in 2014, and access to antenatal care and nutritional supplementation during pregnancy has improved considerably [6]. These improvements may have reduced the biological and social risks traditionally associated with both younger and older maternal ages, buffering their impact on child growth outcomes. The adverse effects of young or advanced maternal age may be more pronounced in settings with more limited health care access.

Second, family and community support systems in Indonesia may help buffer some caregiving challenges faced by younger parents. The extended family structure common in many Indonesian communities, particularly in rural areas, may provide practical and emotional support that helps younger mothers navigate child-rearing challenges, thereby reducing the long-term impact on child growth outcomes. In contrast, settings where nuclear family structures predominate or where support systems are weaker may show stronger associations between young maternal age and child undernutrition.

Third, Indonesia’s family planning programs have been successful in reducing high-risk fertility behaviors. The prevalence of high-risk births declined from 42.8% in 1991 to 29.7% in 2017 [13] reflecting increased use of contraceptives and improved reproductive health services. This may have shifted the distribution of maternal age and parity toward lower-risk categories, reducing the population-level impact of these factors on child stunting.

Fourth, the nonsignificant effect of parity may indicate that the number of children alone is not the primary mechanism influencing stunting risk. Rather, its effect may be partly mediated through birth spacing, as women with higher parity are more likely to experience shorter IPIs. The independent contribution of parity was attenuated once the birth interval was included in the model, showing that inadequate maternal recovery between pregnancies may be more critical than family size itself. This pattern has also been observed in other studies where the effect of parity was explained by the spacing of births [18,47].

These findings do not necessarily contradict the “Four Too” framework but instead suggest that its components may differ in their relative importance for specific child health outcomes and across different contexts. Although maternal age and parity remain relevant for broader maternal and reproductive health outcomes in Indonesia, our findings indicate that short birth intervals may represent the most immediate and modifiable risk factor for stunting in the Indonesian context. This highlights the importance of context-specific research to inform targeted interventions, rather than assuming that the relative importance of risk factors is universal across settings.

This study contributes to the existing literature by providing longitudinal evidence from Indonesia, a country with one of the highest stunting burdens in Southeast Asia. Although cross-sectional studies have documented the association between short birth intervals and stunting, our findings extend this evidence by demonstrating that the adverse effects may persist from early childhood into adolescence. These findings highlight that birth spacing is not only a reproductive health issue but also an important component of nutrition-sensitive strategies, as it influences maternal nutritional recovery, infant feeding practices, caregiving quality, and household resource allocation. Strengthening integrated interventions that combine family planning, maternal nutrition, and child health services may provide sustainable benefits for stunting reduction. Although this study represents an incremental contribution, it offers important context-specific longitudinal evidence to inform Indonesia’s policy and program design.

This study has several strengths. First, a retrospective cohort design was employed using longitudinal data from the Indonesian International Fisheries Service, which provides comprehensive and robust information across multiple waves and regions in Indonesia. The use of longitudinal data enables the examination of high-risk births and their long-term impacts on stunting from early childhood to adolescence, allowing for a more nuanced understanding compared with cross-sectional studies that are often limited to specific age groups. The inclusion of a large and nationally representative sample increases the generalizability of the findings to the Indonesian population. Furthermore, the longitudinal design and multilevel analytical approach allowed for a more robust assessment of the association between high-risk fertility behaviors and stunting while accounting for repeated measurements and clustering effects. These strengths provide valuable evidence on the long-term consequences of high-risk fertility behaviors for child growth and nutritional outcomes in Indonesia.

The findings of this study have reasonable generalizability to the Indonesian context, as the IFLS employs a multistage stratified random sampling design covering over 80% of Indonesia’s population across 24 provinces. The sample includes diverse geographic areas (including rural and urban settings) and socioeconomic strata, thereby enhancing external validity. However, certain limitations to generalizability should be noted. This study was conducted in Indonesia, a middle-income country with a specific cultural and demographic context; therefore, the results may not be directly generalizable to other low- and middle-income countries with different health systems, nutritional patterns, and family planning practices. In addition, the study period (2000–2014) may not fully reflect current conditions, given recent improvements in stunting prevalence and changes in health care access. Nevertheless, the consistent association between short birth intervals and stunting across diverse subpopulations in Indonesia suggests that this relationship is robust and likely relevant to other similar settings. Future studies should examine the generalizability of these findings to other Southeast Asian countries and beyond.

Despite these strengths, this study has several limitations. First, although the IFLS provides extensive longitudinal information, it does not provide detailed data on maternal health and nutritional status during pregnancy, including maternal anemia, micronutrient deficiencies, dietary intake, gestational weight gain, and other pregnancy-related complications. These factors are recognized determinants of fetal growth and child nutritional outcomes and may have influenced the observed associations between high-risk fertility behaviors and stunting.

Second, the exclusion of preterm and small-for-gestational-age (SGA) infants, while methodologically justified to reduce heterogeneity in growth outcomes and ensure appropriate application of World Health Organization (WHO) growth standards, may affect the generalizability of our findings. Preterm and SGA infants are at higher risk of subsequent growth faltering and may be more common among parents with short birth intervals, potentially reflecting maternal nutritional depletion and incomplete physiological recovery between pregnancies [36]. Preterm birth and SGA may function as intermediate mechanisms linking short birth intervals to later stunting. Therefore, excluding these groups may have resulted in conservative effect estimates by removing a particularly vulnerable subgroup in whom the association between short birth intervals and stunting might be even stronger.

To evaluate the robustness of our findings, a sensitivity analysis was conducted, including preterm and SGA infants (n = 1683). The association between short birth interval and stunting remained consistent (odds ratio [OR] = 1.58; 95% confidence interval [CI]: 1.15–2.18), suggesting that these exclusions did not materially alter the main findings. However, the prevalence of stunting was higher in the sensitivity analysis (42.3% vs. 37.7% at baseline), reflecting the greater vulnerability of these groups. Therefore, although our main findings are robust, they may underestimate the true burden of stunting in the general population and the strength of associations among the most vulnerable subgroups.

Future studies should consider including preterm and SGA infants with appropriate age correction and stratified analyses to better understand the distinct pathways to growth impairment in these populations. In addition, the use of longitudinal growth monitoring with more frequent measurements would allow for more detailed characterization of growth trajectories and better identification of critical windows for intervention.

Third, information on pubertal development was excluded from the dataset. Because puberty is a critical determinant of linear growth during adolescence, the absence of pubertal indicators may have affected the interpretation of height trajectories and stunting status among older participants. Moreover, several behavioral and dietary variables were unavailable for younger children because the relevant IFLS questionnaire modules were only administered to respondents aged 15 years. The potential influence of individual dietary practices, smoking behavior, and other lifestyle factors on growth outcomes during childhood could not be comprehensively assessed.

Fourth, data on complementary feeding practices were limited, preventing a detailed evaluation of the quality, diversity, timing, and adequacy of complementary foods, which are important determinants of child growth and nutritional status. Fifth, although household sanitation and drinking water variables were included in the analysis, the dataset lacked more comprehensive measures of environmental exposures, such as household air pollution, water contamination, and other environmental health risks that may contribute to growth faltering.

Sixth, approximately 9.1% of participants were excluded due to incomplete information on anthropometric measurements or key study variables. Although this proportion was below the commonly accepted threshold for concern and complete-case analysis was considered appropriate, residual selection bias cannot be excluded. In addition, the relatively long interval between survey waves (seven years) limited our ability to identify the precise timing of transitions in growth faltering, catch-up growth, and stunting status throughout childhood and adolescence. Finally, as with all observational studies, causal inferences should be made with caution. Despite adjustment for a broad range of children, parental, household, and environmental characteristics, residual confounding from unmeasured factors cannot be completely ruled out.

Nevertheless, this study has several important strengths. The study used data from the Indonesia Family Life Survey, one of the largest and most comprehensive longitudinal population-based surveys in low- and middle-income countries. The availability of repeated anthropometric measurements over a 14-year follow-up period enabled the examination of long-term growth trajectories from early childhood to adolescence.

It is important to consider the temporal context of this study, as the final wave of data was collected in 2014. While this historical cohort provides valuable longitudinal insights into the long-term effects of birth spacing on stunting, several changes in Indonesia’s health care landscape between 2014 and 2026 should be considered when interpreting the findings. Indonesia has made significant progress in stunting reduction, with the prevalence declining from 37.2% in 2013 to 21.6% in 2022 [5]. The launch of the National Strategy for Accelerating Stunting Prevention (2018–2024) and the expansion of the National Health Insurance program (Jaminan Kesehatan Nasional—JKN) have improved access to maternal and child health services. In addition, the ongoing strengthening of community-based health programs (Posyandu) and the increasing coverage of family planning services may have reduced the prevalence and associated risks of short birth intervals. However, the persistent challenges of high stunting prevalence, regional disparities, and the impact of the COVID-19 pandemic on health services and nutrition programs imply that our findings remain relevant for informing current policy discussions. In this cohort, the observed long-term effects of birth spacing are likely to persist, even as the overall prevalence of stunting declines. Therefore, our findings underscore the continued importance of promoting optimal birth spacing as part of comprehensive stunting prevention strategies, which remains relevant to Indonesia’s current goal of achieving a 14% stunting prevalence target. Future research using more recent data is needed to confirm the sustained effects of SBIs in the context of evolving health care policies.

## 5. Conclusions

In conclusion, this study provides evidence that closely spaced births (intervals of 24 months) in Indonesian children are associated with an increased risk of stunting, with effects that persist from early childhood through adolescence. Our findings suggest that even after accounting for a comprehensive range of potential confounders, a short birth interval is a significant determinant of chronic undernutrition. However, the association between other high-risk fertility behaviors (maternal age and parity) and stunting was not statistically significant. This discrepancy may reflect improvements in maternal and child health services, changing socioeconomic conditions, or differences in study populations and methodologies.

The findings underscore the potential importance of strengthening maternal and child health programs, particularly by enhancing access to and quality of postpartum family planning services. Educational interventions targeting parents and families regarding the benefits of maintaining optimal birth intervals of at least 24 months may reduce stunting risk. However, given the observational nature of this study and its limitations, these findings should be interpreted with caution. Future research using more recent data, including measures of maternal nutritional status during pregnancy and detailed dietary information, is needed to further elucidate the causal pathways linking birth spacing to stunting. Intervention studies are warranted to evaluate the effectiveness of family planning programs in reducing stunting prevalence in Indonesian settings.

## Figures and Tables

**Table 1:** Child characteristics (0–59 months) based on IFLS 2000 data (n = 1401).

Variable	n (%)
Sex	
Male	741 (52.9)
Female	660 (47.1)
Age (months)	
<6	268 (19.1)
6–11	260 (18.6)
12–24	462 (33.0)
25–36	244 (17.4)
37–59	167 (11.9)
Birth Weight	
Normal (≥2500 g)	1324 (94.5)
Low Birth Weight (LBW) (<2500 g)	77 (5.5)
Preterm Birth	
No	1275 (91.0)
Yes	126 (9.0)
Early Initiation of Breastfeeding	
Yes	1374 (98.1)
No	27 (1.9)
Exclusive Breastfeeding	
Yes	128 (9.1)
No	1273 (90.9)
Health History	
Diarrhea	249 (17.8)
Acute respiratory tract infection (ARI)	976 (69.7)
Infectious disease (e.g., fever)	1079 (77.0)
Outpatient Visits	
No	964 (68.8)
Yes	437 (31.2)
Delivery Assistance	
Obstetrician	148 (10.6)
Midwife	724 (51.7)
Traditional Birth Attendant/Other	529 (37.8)
Delivery Facility	
Government Hospital	98 (7.0)
Private Hospital	74 (5.3)
Maternity Clinic	447 (31.9)
Community Health Center	66 (4.7)
Home/Traditional Attendant	716 (51.1)

**Table 2:** Children’s characteristics in 2007 and 2014 (n = 1401).

Variable	IFLS 2007	IFLS 2014
Food consumption score		
Poor (<35)	260 (18.6%)	509 (36.3%)
Good (≥35)	1 141 (81.4%)	892 (63.7%)
Physical activity		
Adequate	314 (22.4%)	607 (43.3%)
Inadequate	1087 (77.6%)	794 (56.7%)
Smoking habits		
No	-	1263 (90.2%)
Yes	-	138 (9.9%)

**Table 3:** Parental characteristics (IFLS 2000, 2007, and 2014).

Variables	IFLS 2000	IFLS 2007	IFLS 2014
Father’s Employment			
Unemployed	22 (1.6)	103 (7.4)	145 (10.4)
Employed	1379 (98.4)	1298 (92.6)	1256 (89.6)
Father’s Education			
High School/College	520 (37.1)	520 (37.1)	520 (37.1)
≤Junior High School	881 (62.9)	881 (62.9)	881 (62.9)
Father’s Smoking			
No	458 (32.7)	405 (28.9)	428 (30.6)
Yes	943 (67.3)	996 (71.1)	973 (69.5)
Mother’s Employment			
Unemployed	882 (63.0)	840 (60.0)	731 (52.2)
Employed	519 (37.0)	561 (40.0)	670 (47.8)
Mother’s Education			
High School/College	436 (31.1)	436 (31.1)	436 (31.1)
≤Junior High School	965 (68.9)	965 (68.9)	965 (68.9)
Maternal Height			
At least 150 cm	616 (44.0)	579 (41.3)	575 (41.0)
<150 cm	785 (56.0)	822 (58.7)	826 (59.0)
Household Characteristics			
Residence			
Urban	637 (45.5)	726 (51.8)	868 (62.0)
Rural	764 (54.5)	675 (48.2)	533 (38.0)
Food expenditure ratio			
<60% (Good)	516 (36.8)	813 (58.0)	1084 (77.4)
≥60% (Poor)	885 (63.2)	588 (42.0)	317 (22.6)
Asset Quintile			
Quintile 1 (Lowest)	309 (22.1)	218 (15.6)	254 (18.1)
Quintile 2	267 (19.1)	278 (19.8)	264 (18.8)
Quintile 3	287 (20.5)	317 (22.6)	284 (20.3)
Quintile 4	275 (19.6)	316 (22.6)	305 (21.8)
Quintile 5 (highest)	263 (18.8)	272 (19.4)	294 (21.0)
Sanitation			
Improved	885 (63.2)	1041 (74.3)	1156 (82.5)
Unimproved	516 (36.8)	360 (25.7)	245 (17.5)
Drinking water source			
Improved	1241 (88.6)	1251 (89.3)	1278 (91.2)
Unimproved	160 (11.4)	150 (10.7)	123 (8.8)

**Table 4:** High-risk births (the “Four Toos”) based on IFLS 2000 data (n = 1401).

Characteristics	n (%)
Maternal Age at Birth (MAA)	
20–35 years	1089 (77.7)
Too Early (<20 years)	178 (12.7)
Too Late (>35 years)	134 (9.6)
Parity	
<4 children	1174 (83.8)
Too Many (≥4 children)	227 (16.2)
Birth Interval	
Minimum of 24 months	1306 (93.2)
Too Close (<24 months)	95 (6.8)
High-Risk Birth Status	
No risk factors	895 (63.9)
At least 1 risk factor	506 (36.1)
Number of Risk Factors	
0	895 (63.9)
1	384 (27.4)
At least 2	122 (8.7)

**Table 5:** Impact of births and the “Four Too” covariates on stunting.

Variables	Null Model	Model 4 (Child Factors)	Model 4a (+Parental)	Model 4b (+Socioeconomic)	Model 4c (+Physical Activity/FCS)	Model 4d (Full)
Fixed effects (OR, 95% CI)						
Intercept	0.43 *** (0.39–0.47)	0.28 *** (0.17–0.45)	0.25 *** (0.14–0.47)	0.29 *** (0.15–0.58)	0.22 *** (0.11–0.45)	0.23 *** (0.11–0.47)
Four Toos						
Maternal age (<20 vs. 20–35 years)	-	1.10 (0.86–1.40)	1.09 (0.86–1.38)	1.01 (0.79–1.30)	1.03 (0.80–1.31)	1.03 (0.81–1.32)
Maternal age (>35 years vs. 20–35 years)	-	0.87 (0.63–1.18)	0.85 (0.63–1.16)	0.88 (0.64–1.21)	0.88 (0.64–1.20)	0.88 (0.64–1.20)
Birth interval (<24 months)	-	1.63 ** (1.21–2.20)	1.63 ** (1.21–2.19)	1.61 ** (1.19–2.19)	1.59 ** (1.17–2.16)	1.60 ** (1.18–2.16)
Parity (≥4)	-	1.18 (0.92–1.52)	1.20 (0.93–1.54)	1.16 (0.90–1.49)	1.15 (0.89–1.48)	1.15 (0.89–1.48)
Child factors						
Age group (months)						
<6	Ref					
6–11	-	1.23 (0.93–1.61)	1.21 (0.92–1.59)	1.20 (0.91–1.59)	1.26 (0.96–1.67)	1.27 (0.96–1.68)
12–24	-	1.93 ** (1.51–2.45)	1.92 ** (1.51–2.44)	1.94 ** (1.52–2.47)	2.11 ** (1.64–2.71)	2.13 ** (1.66–2.75)
25–36	-	2.01 ** (1.53–2.64)	1.98 ** (1.51–2.59)	2.00 ** (1.52–2.62)	2.18 ** (1.64–2.88)	2.21 ** (1.66–2.92)
37–59	-	1.61 (1.19–2.17)	1.57 (1.17–2.12)	1.62 (1.20–2.20)	1.79** (1.31–2.44)	1.81 ** (1.33–2.48)
Female (sex)	-	0.82 ** (0.70–0.96)	0.83 ** (0.71–0.97)	0.84 ** (0.71–0.98)	0.84 ** (0.71–0.98)	0.82 ** (0.69–0.96)
Low birth weight (yes)	-	1.69 (1.20–2.39)	1.61 (1.15–2.27)	1.56 (1.11–2.21)	1.55 (1.09–2.19)	1.55 (1.10–2.20)
Parental factors						
Maternal height (<150 cm)	-	-	1.53 ** (1.31–1.79)	1.50 ** (1.28–1.76)	1.51 ** (1.28–1.77)	1.50 ** (1.28–1.76)
Socioeconomic & environmental Factors						
Unimproved sanitation	-	-	-	1.36 ** (1.14–1.64)	1.34 (1.12–1.60)	1.33 (1.11–1.59)
Food expenditure (>60%)	-	-	-	1.40 ** (1.20–1.65)	1.38 ** (1.17–1.62)	1.37 ** (1.16–1.61)
Residence (urban)	-	-	-	0.77 (0.64–0.91)	0.77 (0.65–0.92)	0.78 (0.65–0.93)
Behavioral factors						
Low physical activity	-	-	-	-	1.23 ** (1.03–1.48)	1.21 * (1.01–1.46)
Smoking	-	-	-	-	-	0.66 ^†^ (0.43–1.03)
Random effects						
Variance (hhid)	0.70	0.40	0.35	0.34	0.30	0.31
Variance (pidlink|hhid)	0.51	0.14	0.13	0.17	0.21	0.22
ICC						
- hhid	0.12	0.10	0.09	0.09	0.08	0.08
- pidlink|hhid	0.19	0.14	0.13	0.14	0.14	0.14
Median odds ratio	2.22	1.83	1.76	1.74	1.69	1.70
Model statistics						
Log likelihood	−2630.38	−2557.69	−2542.31	−2507.68	−2503.43	−2501.67
LR test (χ^2^)	-	134.19 ***	161.12 ***	210.67 ***	216.39 ***	218.62 ***

Notes: *** *p* < 0.001; ** *p* < 0.01; * *p* < 0.05; ^†^ *p* < 0.1; OR = odds ratio; CI = confidence interval; LBW = low birth weight; FCS = food consumption score; ICC = intraclass correlation coefficient; MOR = median odds ratio. Outcome variable: Stunting (LAZ/HAZ < −2 SD) as a binary outcome. Predictor variables are selected according to child, parental, and household factors associated with stunting. The significant predictors in the adjusted model are highlighted in bold.

## Data Availability

This study analyzed publicly available datasets. These data are available from the Indonesia Family Life Survey (IFLS) at https://www.rand.org/health/surveys/FLS/IFLS/access.html (accessed on 16 March 2026), subject to registration and approval of data access requests.

## Abbreviations

The following abbreviations are used in this manuscript:

IFLS	Indonesia’s Family Life Survey
4T	4 (four) Too
WHO	WHO
DHS	The Demographic Health Survey
ARI	Acute infection of the respiratory tract
LBW	Low birth weight
FCS	Food consumption score
HAZ	Height-for-age Z-score
OR	Odds ratio
CI	Confidence interval
ICC	Intraclass correlation coefficient
MOR	Median odds ratio
WASH	Water, sanitation, and hygiene

## Appendix A

**Table A1:** Variables and operational definitions.

Category	Variable	Operational Definition	Measurement Results	Year
Dependent variables	Stunting	Nutritional status based on length for age < –2 SD of the WHO Child Growth Standards median.	1. HAZ < −2 SD 2. HAZ ≥ −2 SD	2000, 2007, and 2014
Main independent variables	Advanced maternal age	Maternal age during childbirth was classified as advanced when the patient was 35 years or older [13]	0: No 1: Yes	2000
	Young maternal age	Maternal age during childbirth is classified as younger than 20 years [13]	0: No 1: Yes	2000
	High parity	Having four or more children.	0: No 1: Yes	2000
	Short birth interval	The birth interval was classified as short (<24 months [13]	0: No 1: Yes	2000
Covariates				
Child characteristics	Child age	Child’s age, calculated as the duration from birth until the interview date.	Categorized as follows: a. 0–5 months, b. 6–11 months, c. 12–24 months, and d. >24 months	2000
	Sex	Biological sex of the child.	0: Male 1: Female	2000
	Preterm birth	Infants born alive before 37 weeks of pregnancy are completed.		2000
	Low birth weight	WHO defines low birth weight as a weight at birth of 2500 g (5.5 pounds).	Numerical	2000
	Exclusive breastfeeding	Breast milk was exclusively given to infants for the first 6 months without supplementary food or drink.	0: Yes 1: No	2000
	Early breastfeeding initiation	Placement of a newborn on the mother’s chest or abdomen immediately after birth to allow the infant to latch onto the breast and start nursing.	0: Yes 1: No	2000
	Acute infection of the respiratory tract (ARI)	Presence or absence of ARI within the past 2 weeks.	0: No 1: Yes	2000
	Diarrhea	Three or more stools that are sufficiently liquid to take the shape of the container in which they are placed in the last 2 weeks are passed in 24 h. Presence and duration of diarrhea in the last 2 weeks.	0: No 1: Yes	2000
	Infection history	Infection defined as a history of cough, diarrhea, or fever in the last 2 weeks (yes, either of the three conditions)	0: No 1: Yes	2000
	Outpatient visit	A visit to a health care facility, such as a clinic, primary care center, or hospital outpatient department, for medical consultation or treatment without an overnight stay. Outpatient visits include scheduled appointments for routine checkups, diagnoses, or follow-up care, as well as walk-in consultations.	0: No 1: Yes	2000
	Birth attendant	Type of professional assistance during childbirth.	1. Obstetricians 2. Midwives 3. Others	2000
	Place of delivery	Location where the respondent gave birth.	1. Government hospital 2. Private hospital 3. Maternity clinic 4. Primary health center	2000
Parental Characteristics	Maternal height	Maternal height was used as an indicator of nutritional status during the growth period, which was assessed through height measurement.	0: >150 cm 1: ≤150 cm	2000, 2007, and 2014
	Maternal Education	The highest education level completed by the mother.	0: No formal education 1: Primary school 2: Secondary school 3: Higher education	2000, 2007, and 2014
	Maternal employment	Mother’s employment status categorized as employed or unemployed.	0: Unemployed 1: Employed	2000, 2007, and 2014
	Paternal education	The parent completed the highest level of formal education, as verified by the certificate.	0: Secondary school and lower 1: Secondary school	2000, 2007, and 2014
	Paternal employment	Primary occupation or activity occupying most of the father’s time, categorized as not working, studying, or employed (government/private sector, entrepreneur, farmer, etc.).	0: No 1: Yes	2000, 2007, and 2014
	Paternal smoking	Whether the parent smoked actively and has smoked for at least one year.	0: No 1: Yes	2000, 2007, and 2014
Household characteristics	Percentage of household food expenditure	Total expenditure on food purchases over the past month, calculated using the following formula: (Total expenditure on food items, including staple foods, vegetables, dried foods, side dishes, seasonings, and other foods)/(Total food expenditure + Non-food expenditure, including electricity, water, fuel, telephone, household needs, recreation, and transportation) × 100%	0: Good (below 60%) 1: Poor (60%)	2000, 2007, and 2014
	Household asset ownership	Household ownership of valuable assets is an indicator of economic capability, which is converted into monetary terms (Indonesian rupiah). These assets include housing, land, vehicles, savings, jewelry, and electronic goods.	The total asset value, converted into rupiah, was categorized into five quintiles.	2000, 2007, and 2014
	Place of residence	The location of the household, categorized by urban or rural area.	0: Rural 1: Urban	2000, 2007, and 2014
	Improved drinking water quality	Improved drinking water sources are defined based on the WHO/UNICEF Joint Monitoring Program (JMP) as those that are likely to be protected from outside contamination, particularly fecal matter. Improved water sources include household connections, public standpipes, boreholes, protected dug wells, protected springs, and rainwater collection. Unimproved water sources include unprotected wells, unprotected springs, surface water (e.g., river, dam, or lake), vendor-provided water, bottled water (unless water for other uses is available from an improved source), and tanker truck-provided water	0: Yes 1: No	2000, 2007, and 2014
	Improved Sanitation	Improved sanitation facilities are defined based on the WHO/UNICEF Joint Monitoring Program (JMP) as those that hygienically separate human waste from human contact. Improved sanitation includes flushing or pour-flushing to a piped sewer system, septic tanks, pit latrines, ventilated improved pit latrines, or pit latrines with slab or composting toilets. Shared or public-use sanitation facilities are not improved. Moreover, flushing or pour-flushing elsewhere, pit latrines without slabs or open pits, bucket latrines, hanging latrines, or open defecation are not considered to be improved sanitation.	0: Yes 1: No	2000, 2007, and 2014

## Appendix B

The minimum required sample size was calculated using the two-proportion hypothesis test formula as follows:

The sample size equation:

*n* = (*Z*_α/2_ + *Z*_β_)^2^ × (*P*_1_(1 − *P*_1_) + *P*_2_(1 − *P*_2_))/(*P*_1_ − *P*_2_)^2^

where:

*n* = minimum sample size (580 × 2 = 1160)
*Z*_1−α_ = *Z* value at the significance level (one-tailed α = 5%; *Z* = 1.64)
*Z*_1−β_ = *Z* value corresponding to the statistical power (1 − β = 90%)
*P*_1_ = proportion of stunting among children born to women with one component of the “Four Too” high-risk fertility behavior (44.2%) [49]
*P*_2_ = proportion of stunting among children born to women with more than one component of the “Four Too” high-risk fertility behavior (53.7%) [49]

Based on this calculation, the minimum required sample size was 1160 respondents.

## Appendix C

Appendix C and Table A2: Stunting Status Transition Across IFLS Waves.

**Table A2:** Individual-level transitions in stunting status between 2000, 2007, and 2014 (n = 1401).

Transition Category	2000–2007 n (%)	2007–2014 n (%)	2000–2014 n (%)
Remained normal	688 (49.1)	912 (65.1)	601 (42.9)
Height faltering (normal → stunted)	168 (12.0)	132 (9.4)	145 (10.4)
Catch-up growth (stunted → normal)	238 (17.0)	174 (12.4)	356 (25.4)
Remained stunted	307 (21.9)	183 (13.1)	299 (21.3)
Total	1401 (100)	1401 (100)	1401 (100)

Footnote: Stunting status was classified using age-standardized height-for-age z-scores (HAZ) based on the World Health Organization Child Growth Standards (for children aged 0–5 years) and the World Health Organization Growth Reference (for children aged 5–19 years). The transition categories were as follows: remained normal (normal at both waves), height faltering (normal to stunted), catch-up growth (stunted to normal), and remained stunted (stunted at both waves).

Supplementary transition analyses (Appendix C, Table A2) showed that stunting status was dynamic across childhood and adolescence. Between 2000 and 2007, 21.9% of children remained stunted, while 17.0% experienced catch-up growth. Between 2007 and 2014, the proportion of children with persistent stunting declined to 13.1%, although 9.4% experienced height faltering. Across the full follow-up period (2000–2014), 21.3% of patients remained stunted, whereas 25.4% achieved catch-up growth. Although some recovery occurred during adolescence, a substantial proportion of children experienced persistent or recurrent growth faltering.
